# Remediation Technology and Typical Case Analysis of Informal Landfills in Rainy Areas of Southern China

**DOI:** 10.3390/ijerph17030899

**Published:** 2020-01-31

**Authors:** Qin Yin, Haihong Yan, Xiaoya Guo, Yu Liang, Xingzhi Wang, Qian Yang, Shuqi Li, Xianqi Zhang, Yuexi Zhou, Yuegang Nian

**Affiliations:** 1 College of Water Science, Beijing Normal University, Beijing 100875, China; 2 State Key Laboratory of Environmental Criteria and Risk Assessment, Pollution Control Research Center, Chinese Research Academy of Environmental Science, Beijing 100012, China; 3 Academy of water resources and environment, China University of Geosciences, Beijing 100083, China

**Keywords:** informal landfill, rainy area, investigation, environmental quality survey, remediation technology

## Abstract

A typical informal landfill in a rainy area of southern China was taken as an example in this study. The comprehensive control ideas and processes of the informal landfill site were systematically reviewed. The basic situation for the early stage of the government survey and investigation was provided, including a waste stock survey, water volume measurement, and a waste source survey. The main contents and key factors of a comprehensive investigation of the environmental quality status were briefly summarized. The water quality in the landfill, groundwater quality inside and outside of the site, and heavy metals in the bottom sediment were all determined. A low-cost practical landfill technology was explored to reduce the Chemical Oxygen Demand COD_Cr_ concentration of polyaluminum ferric chloride (PAFC), and NH_4_^+^-N was removed by calcium hypochlorite. Soil backfill was replaced, such that the informal landfill site was immobilized, which was perfectly suitable for this southern rainy area. This study proposes rules for a comprehensive improvement scheme for a landfill, and provides a reliable theoretical basis and practical experience for the treatment of similar informal landfills.

## 1. Introduction

Informal landfills refer to sites that use natural terrain conditions for waste treatment and are not constructed and operated in accordance with relevant national standards and norms [1,2]. They are characterized by unsophisticated construction, lack of environmental protection measures, and a high risk of environmental pollution. They are considered informal landfills because of the lack of a sound seepage control system and garbage leachate collection and treatment facilities. The leachate from the landfill sites poses a serious threat to the surrounding groundwater environment [3], particularly for the high rainfall region of southern China, with its shallow groundwater level. Ammonia, chloride, heavy metal ions, and other organic compounds in leachate are released into the environment, and their toxicity makes leachate potentially hazardous to the environment [4,5,6,7].

Low-lying landfills form pools where waste is subject to long-term immersion, increasing the leaching efficiency of pollutants. Heavy seasonal rains and subsequent water flow causes surrounding surface water and soil environment pollution [8,9,10].

Given the continuous updating of national environmental protection policies and the improvement of public environmental protection awareness during recent years, the governance of informal landfills has become an imperative environmental protection task [11]. Because the construction, use, and operation of informal landfill sites differs from that of sanitary landfill sites, investigation before implementation of governance work and a targeted scheme design are particularly important [12].

At present, China has accumulated a relative breadth of experience in the comprehensive treatment of informal landfills, but there is still a lack of systematic management ideas and complete technical schemes for informal landfills in rainy areas in southern China. The main reason for this is that for this type of landfill, during the process of treatment, in addition to considering the treatment and disposal of solid waste, there is also the problem of water environmental treatment as a result of rainfall. At the same time, the waste is degraded and converted to silt, which causes insufficient stability of its structure during the comprehensive treatment process and affects the implementation of the project. Silt pollutants result in questions of how to safely and effectively control or treat waste, which will influence the final determination of regulation schemes.

There are few reports regarding the treatment of water accumulated at informal landfills, but research regarding leachate in sanitary fields is quite mature and has achieved good results. Leachate contains a large number of harmful organic matters, heavy metals, and inorganic salts [13]. There are many means to treat leachate, such as membrane bioreactors [14] adsorption [15], flocculation [16,17], and electrochemical methods [18]. Advanced oxidation techniques are a good way to treat leachate, such as fenton, ozone, or other improved methods [19,20,21,22,23,24].

Treatment of leachate in general sanitary landfills is conducted in the field; however, this type of long process technology is not suitable for informal landfills. Leachate is treated only once, that is, after treatment, water is removed or connected to a municipal pipe network and no subsequent leachate is generated. At present, there is no effective integrated equipment to sufficiently treat leachate. Mature, low-cost, and high-efficiency leachate treatment technology is an effective means to solve the problem of water management at informal landfills. Regarding site investigations of informal landfills, most focus on the site risk assessment of the landfills, and those in arid areas of northern China are often taken as case studies [25].

A survey of this type in the rainy areas in southern China has not been previously reported. Taking an informal landfill site in southern China as a typical case, this study aimed to (1) select the specific method of basic situation for the early stage of the government survey and investigation; (2) determine the main contents and key factors of comprehensive investigation of the environmental quality status; (3) explore a low-cost practical landfill technology to solve water pollution problem in site; and (4) summarize the processes of comprehensive treatment of the informal landfill site in a rainy area of southern China.

## 2. Materials and Methods

### 2.1. Project Area Overview

**Figure 1:**
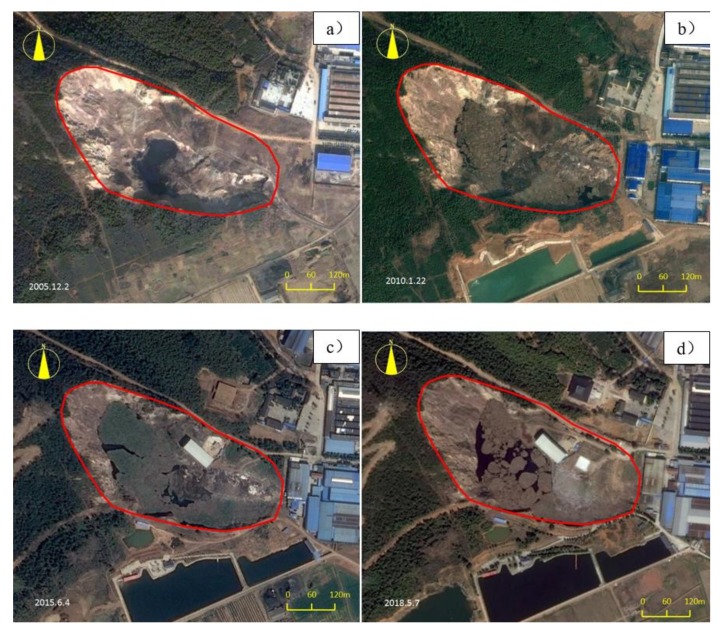
Historical Google satellite images of landfills: (**a**): 2005; (**b**): 2010; (**c**): 2015; (**d**): 2018.

The informal landfill is in southern China, with an average annual rainfall of 1034.4 mm, mainly from June to September, and an approximately 226-day frost-free period. The site was previously an abandoned quarry. As a result of years of mining, a natural pit formed. Beginning in the 1990s, waste began to accumulate in the pit. The changing process of the landfill is shown in Figure 1. It can be seen from the Google satellite images that the water in the landfill continuously increased from 2005 to 2015. In 2018, the local government took measures to establish a waste salvage platform and preliminarily cleaned it up.

### 2.2. Site Status Survey

#### 2.2.1. Waste Stock Measurement

**Figure 2:**
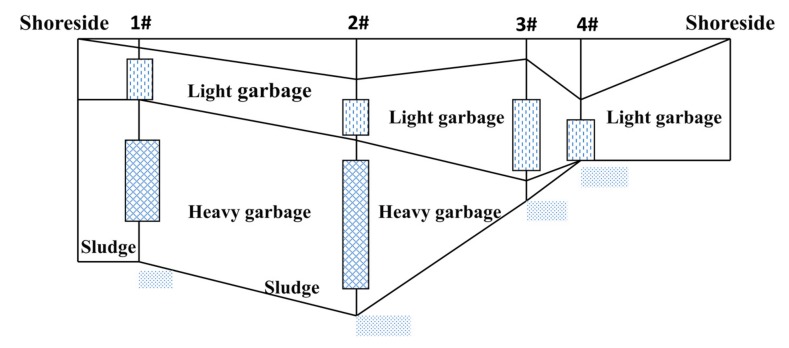
Geophysical profile.

As the waste stock in the landfill is not clear, we used a method of geophysical exploration to determine the hydrology in the field. We drilled holes at positions 1–4 to determine water depth, garbage depth and silt depth. The range finder was used to measure the size of the site. The geophysical profile is shown in Figure 2. By the calculation, the informal landfill covers an area of approximately 28,000 m^2^, the stock of waste and mudstone is approximately 240,000 m^3^, and the leachate volume is approximately 30,000 m^3^. The water depth is up to 12 m.

#### 2.2.2. Waste Source Survey

**Figure 3:**
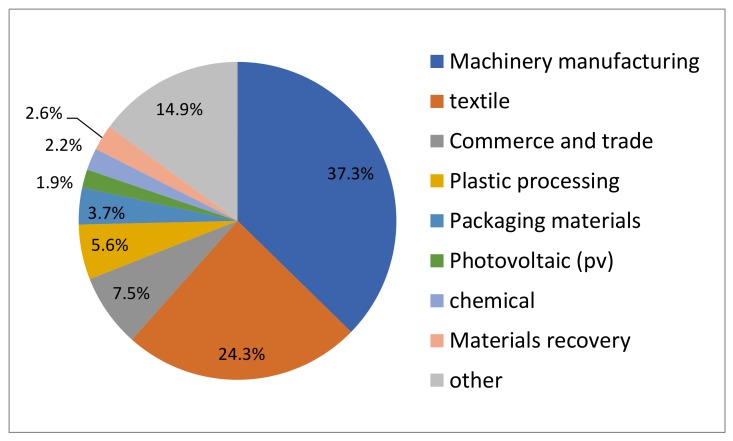
Classification and analysis of industrial enterprises around the landfill during the past 30 years.

The investigation of the source of the waste is a necessary to determine the types of pollutants at the site. For this informal landfill, it is known that the waste in the site mainly consists of textile industrial waste and domestic waste through public inquiry and a data search. The classification and analysis of the industrial enterprises around the landfill during the past 30 years is shown in Figure 3. The number of enterprises is 142, mainly involving machinery manufacturing and textile and commerce trade; these three types of enterprises account for 37.3%, 24.3%, and 7.5% of the waste, respectively. In this investigation, through obtaining the registration information of the local industry and Commerce Department, it was found that the waste generated by the local chemical industry was collected by qualified companies. This site does not contain hazardous waste from the medical treatment, chemical, or other industries.

#### 2.2.3. Hydrogeological Survey

The purpose of the hydrogeological survey was to understand the stratigraphic structure of the site and the groundwater level and flow direction, with a focus on determining the possible means by which the site water could contaminate the groundwater.

**Figure 4:**
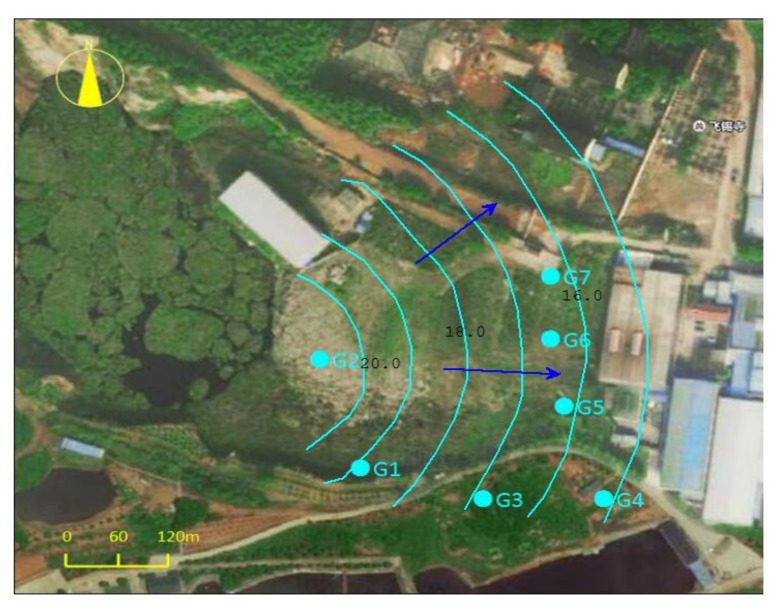
Water level in a contour map of the landfill.

A total of seven drilling wells, G1, G2, G3, G4, G5, G6, and G7, were established in the landfill area. A summary of the measured water level is shown in Table 1. The hole depths of G1–G7 are 4–10 m, the surface elevations are 15–23 m, and the water levels are 0.3–6.7 m. There is no groundwater in G5 and G6, and the water level elevation is 15–22 m. The water level in a contour map of the landfill is shown in Figure 4. It is known that the groundwater flow direction of the site is from southwest to northeast.

### 2.3. Site Environmental Quality Survey

#### 2.3.1. Analysis of Sediment

Considering site subsequent reuse and environment risk analysis, the heavy metals in the sediment needed to be tested. Sediment samples, after being dry ground, were sifted through 100 mesh sieve. The samples (0.1g) were collected and put in PTFE test tube and then nitric acid (5 mL), hydrofluoric acid (2 mL), and perchloric acid (1 mL) were added. The samples were heated at 180℃ for 4h, and then the acid was evaporated. After cooling, the samples were filled to 25 mL with high purity water and detected by inductively coupled plasma atomic emission spectrometry (ICP-AES, SPECTRO Analytical Instruments GmbH, Kleve, Germany). Organic matter content is among the important indicators. A stability assessment field detection method with reference to the determination methods of soil organic matter (NY/T 1121.6 2006) was determined.

The sampling points were geophysical detection points, as shown in Figure 1 (1#, 2#, 3#, and 4#).

#### 2.3.2. Analysis of Water Quality at the Site

**Figure 5:**
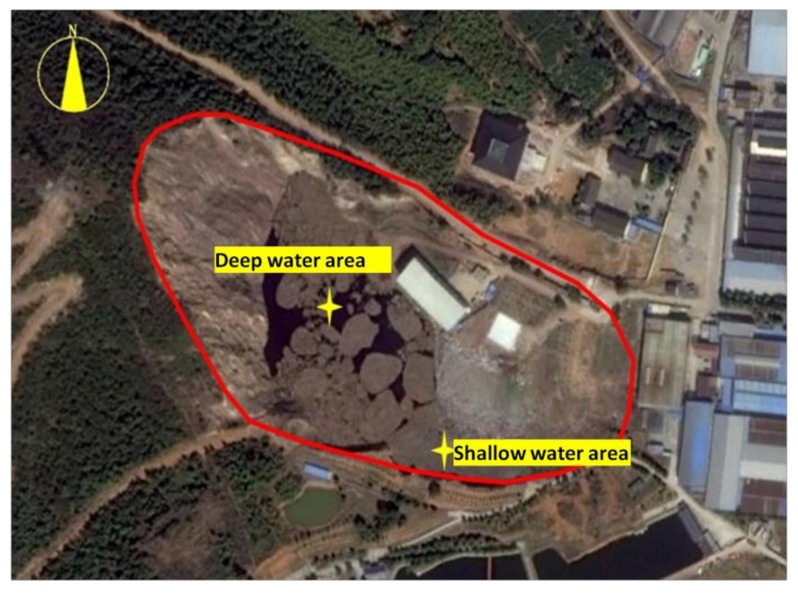
Water collection points in the landfill.

The purpose of the water quality analysis was to understand the water quality of the site so as to determine the subsequent treatment and discharge of water. The main pollutants in the landfill leachate are NH_4_^+^-N and COD_Cr_, and contain indexes that inhibit microbial growth. According to the depth of the landfill, two sampling areas were established in the site as shown in Figure 5. Two samples were collected in the deep-water area, respectively, 0.5 m below the surface (signed DB-0.5) and 0.5 m above the bottom (signed DA-0.5), and one sample was collected in the shallow water area, 0.5 m below the surface (signed SB-0.5). According to the environmental quality standards for surface water [26] and comprehensive sewage discharge standards [27], 16 types of conventional indicators such as ORP, pH, NH_4_^+^-N, COD_Cr_, TP, TN, and sulfide were tested and analyzed. Water quality testing methods are shown in Table 2.

#### 2.3.3. Analysis of Groundwater Quality around the Site

To determine the environmental quality of the groundwater at the landfill site and assess whether the surrounding groundwater environment had been affected by the landfill, groundwater sampling sites were established inside and outside the site for water quality analysis. The sampling sites were G3 (outside the site) and G2 (inside the site) as part of the geological condition detection sites.

Because there is an industrial plant on the east side of a nearby factory, to avoid interference of monitoring point data caused by external factors and lack of conviction, the off-site groundwater monitoring point was set on the south side of the factory, and the testing indicators were analyzed by referring to representative indicators in the groundwater environmental quality standard [28].

## 3. Results and Discussion

### 3.1. Contamination Detection of Sediment in the Yard

The bottom mud in the pit of the landfill site was collected for detection. The detection results are shown in Table 3. As can be seen from the data in the table, the organic matter content of the bottom mud in the pit is 1.72–7.64%, indicating that the waste has reached a certain degree of maturation. According to the risk screening value of the second type of land in the standard for soil environmental quality construction land (trial) [29], all the testing indexes meet the risk screening value requirements (signed soil standard). This indicates that there is no pollution of heavy metals at the site.

### 3.2. Conventional Pollutants in Water

Through testing of the raw water quality of the site, it was found that the COD_Cr_ and NH_4_^+^-N concentration in the pond reached 3012 mg·L^−1^ and 1014 mg·L^−1^, respectively. After surface aeration treatment, the concentration of COD_Cr_ and NH_4_^+^-N in the pond significantly decreased. The results for conventional pollutants in the water are shown in Table 4. The highest concentrations of COD_Cr_, NH_4_^+^-N, and TN were 238 mg·L^−1^, 129 mg·L^−1^, and 148 mg·L^−1^, respectively, exceeding the surface water environment quality standard (v class) [26] and the standard of the sewage comprehensive discharge (Grade I) [27] limit. Fluoride, anionic surfactants, and total phosphorus were slightly greater than the surface water environment quality standard (v class) [26] but met the integrated wastewater discharge standard (Grade I) [27] limit.

It can be concluded that the water in the landfill should be removed or discharged after subsequent treatment to meet the relevant standards. The key pollutant indexes in the water are COD_Cr_, NH_4_^+^-N, and TN.

### 3.3. Comparative Analysis of Groundwater Detection Results Inside and Outside the Site

The results were compared to the IV grade standard for groundwater quality (signed GW-standard) (shown in Table 5). It was found the water is alkaline, and some indicators were outside the limits at the G2 site, including smell and taste, visibility to the naked eye, turbidity, pH, ammonia nitrogen, volatile phenol, and oxygen consumption. Some researchers [7,30] used Cl^-^, NH_4_^+^, Na^+^, and COD as parameters to measure the penetration of landfill leachate pollution. In this study, the excessive turbidity was up to 1049 times the limit; NH_4_^+^-N exceeded the limit by approximately 9 times, and volatile phenol and oxygen consumption exceeded the limit by 7.1 and 1.9, respectively. The three indicators at G3, smell and position, visible objects, and turbidity, exceeded the standard; the excess multiple of turbidity was 2.1. According to the analysis of the G2 data, the groundwater at the site is affected by landfill leachate. By comparing and analyzing the detection data of G2 and G3, it was found that the number of indicators and pollutants exceeding the limits outside the site were far lower than those inside the site, indicating that the environmental quality of the off-site groundwater is less affected by the landfill leachate. The reasons for this may be that the flow direction of the groundwater varies in a certain area because of the microgeomorphic units in southern China and there is no connectivity between G3 and the landfill. This field shows such results, but if the water outside the site is also polluted, then the subsequent site treatment measures will increase the groundwater remediation. For example, vertical curtain or cutoff wall should be adopted to cut off the migration of pollution source to the downstream direction of groundwater flow [31].

### 3.4. Measurements

#### 3.4.1. Waste Salvage

Because all the pollutants at the site originate from the site waste, the primary task to reduce the environmental risk of the landfill is to eliminate the source of pollution. The removal of waste can effectively control the continuous release of pollutants and reduce the environmental risk.

#### 3.4.2. Water Treatment

According to the water quality test results, the main concept of water treatment is to determine a low-cost and efficient water-treatment technology and discharge after meeting the relevant requirements or connect to off-site treatment facilities. Water treatment is the key to the management of this type of landfill. Compared to biochemical treatment, physical-chemical treatment is simple and feasible; thus, it has some advantages in the treatment of water in an informal landfill. At the same time, sewage treatment can be combined with local actual conditions. Sewage can be treated to meet the standards and then removed via a nearby sewage pipe network that can not only reduce the treatment cost but also reduce the environmental risk caused by sewage outflow. Polyaluminum ferric chloride (PAFC) is an inorganic polymer flocculant developed on the basis of the coagulation and hydrolysis mechanism of aluminum salt and iron salt with a good pollutant removal effect. The mixing ratio of the three gradients of 0.4‰, 0.7‰, and 1‰ was selected for a coagulation and precipitation experiment. The results showed that the removal rate of COD_Cr_ could be increased to 35.94% when the ratio of PAFC was 1‰, as shown in Table 6.

Under the condition of this proportion, the COD_Cr_ effluent could meet the requirements of the connection to a sewage pipe network (≤500 mg·L^−1^), but the NH_4_^+^-N concentration could not be met. Thus, calcium hypochlorite (CH) was considered to reduce the NH_4_^+^-N concentration.

**Figure 6:**
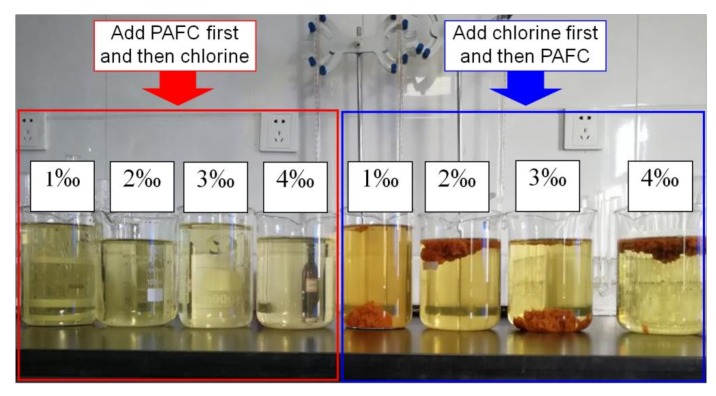
Effect diagram of different dosing sequence.

**Figure 7:**
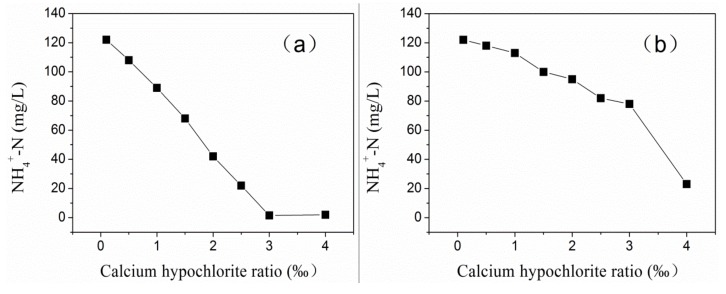
Comparison diagram of the NH_4_^+^-N removal effect in different dosing sequences. (**a**) Add PAFC first and then Chlorine; (**b**) Add Chlorine first and then PAFC.

The proportion of addition was set as 1‰, 2‰, 3.0‰, and 4.0‰ (the effective rate of CH was 50%). Through experimental comparison, it was found that adding PAFC first and then adding CH had a better NH_4_^+^-N removal effect than adding CH first and then adding PAFC. The difference in the effect is shown in Figure 6. The change in the NH_4_^+^-N concentration is shown in Figure 7.

According to the experimental comparison between Figure 6 and Figure 7, adding PAFC first and then the CH treatment results in higher clarity and lower color, better appearance of the treated water, and a faster removal rate of ammonia nitrogen. The reason may be that the later addition of PAFC makes ferric ions free in the reaction phase, which makes the solution appear brownish red. Under the condition that the CH dosage is 3‰, the removal rate of NH_4_^+^-N reaches an optimal state. However, the removal effect of NH_4_^+^-N was relatively poor when the reagent was added in the opposite order. It can be seen that the sequence of adding agents is different, resulting in the difference of reaction process. Under the condition that the same treatment effect is achieved, the amount of adding agents will also change accordingly.

By adopting the treatment method of adding PAFC first and CH later, the sewage in the pond can be treated to the standard and then connected to the downstream sewage plant for further treatment, thus solving the problem of sewage treatment.

#### 3.4.3. Backfilling Guest Soil

After the sewage is discharged, the silt moisture content at the bottom of the pond is very high, which will lead to further expansion of the silt and the formation of surface runoff given the rainwater during the rainy season, endangering the surrounding environment.

It is necessary to immobilize the silt in the field and then stabilize the landfill. In a backfill site with low moisture content, the silt is concentrated into several internal sections of the site and lime is solidified on the surface. Compared to full-field curing, this method can greatly improve the infrastructure stability and lower costs. Through the implementation of this solidification project by backfilling the guest soil, the landfill can meet follow-up stability requirements.

#### 3.4.4. Landfill Covering

The stability of the landfill was greatly improved after the backfilling of the guest soil and lime solidification in the field. The landfill covering was conducted in accordance with the Technical Specification for Sanitary Landfill Covering [32]. This project involved the construction of an impermeable structure layer in the field as an example to determine the main contents of the landfill cover project. The system structure included gas-venting, impermeable, drainage, and vegetation layers.

## 4. Conclusions

Through the investigation and analysis of the current situation of a typical informal landfill in southern China and the detection of the environmental quality of the site, the landfill stock and water volume were determined. The source and properties of incoming waste were determined, and the hydrogeological conditions, water quality, heavy metal content in the bottom mud, and extent of pollution were obtained. It was found that the number of indicators and pollutants exceeding the limits outside the site were far lower than those inside the site, indicating that the environmental quality of the off-site groundwater is less affected by the landfill leachate. However, if the water outside the site is also polluted, then the subsequent site treatment measures will increase the groundwater remediation. Adopting the treatment method of adding PAFC first and CH later results in higher clarity and lower color, better appearance of the treated water, and a faster removal rate of ammonia nitrogen. The sewage in the pond was treated to the standard and then connected to a downstream sewage plant for further treatment, thus solving the problem of sewage treatment. The project of backfilling guest soil and landfill covering fully achieved the comprehensive regulation of the landfill and reduced the risk of the site. The research and application of this project can provide a theoretical basis and practical experience for the comprehensive regulation of informal landfills in rainy areas in southern China.

## Figures and Tables

**Table 1:** Summary of measured water level. Unit: m.

Hole No.	Hole Depth (m)	The Ground Elevation (m)	Water Depth (m)	The Water Level Elevation (m)
G1	10.0	20.6	3.5	17.1
G2	4.0	22.1	0.4	21.7
G3	7.0	19.0	6.4	12.6
G4	13.0	15.5	0.3	15.2
G5	9.0	18.5	No groundwater observed	-
G6	6.5	19.0	No groundwater observed	-
G7	9.0	22.6	6.7	15.9

**Table 2:** Water quality testing methods.

The Index	Test Instrument\Method
ORP\pH	pH and ORP meter
NH_4_^+^-N	Ultraviolet spectrophotometer
COD_Cr_	Rapid digestion spectrophotometry
TP	Molybdenum-antimony resistance spectrophotometry
TN	Total organic carbon analyzer
Fe\Mn\Cu\Cd\Pb\Hg\As\Na\Zn	ICP-AES
Benzene\methylbenzene	GC-MS
Turbidity degree\Volatile Penol\LAS\sulfide	Spectrophotometry
Cl^−^\SO_4_^2−^	Ion chromatography
Total hardness	EDTA titration method
TDS	Sensory traits and physical indicators

ORP: Oxidation-Reduction Potential; TP: Total Phosphorus; TN: Total Nitrogen; ICP-AES: Inductively Coupled Plasma Atomic Emission Spectrometry; GC-MS: Gas Chromatography-Mass Spectrometer; LAS: Anionic Surfactant; TDS: Total Dissolved Solids.

**Table 3:** Bottom sediment test results. Unit: mg·kg^−1^.

Sample Number	Cu	Ni	Hg	Pb	As	Cd	Organic Matter
4#-1	58.4	124	0.096	122	11.6	0.19	29.8
4#-2	19.9	32.2	0.063	15.2	9.7	0.16	48.8
5#-1	28.1	39.4	0.056	18.8	16.4	0.27	76.4
2#-1	67.7	18.5	0.081	10.7	10.9	0.14	17.2
2#-2	30.9	20.3	0.07	17	10	0.26	23.4
1#-1	37.8	34.7	0.089	20.1	7.94	0.19	23.3
Soil standard	18000	900	38	800	60	65	—

**Table 4:** Results of pollutant detection in water.

The Sample Name	ORP	pH	NH_4_^+^-N	COD_Cr_	TP	TN	Sulfide	Benzene	Methylbenzene	SO_4_^2−^
	MV	-	mg·L^−1^	mg·L^−1^	mg·L^−1^	mg·L^−1^	mg·L^−1^	mg·L^−1^	mg·L^−1^	mg·L^−1^
DB-0.5	143	7.91	129	238	0.26	148	ND	ND	ND	64.9
DA-0.5	140	7.95	116	225	0.25	132	ND	ND	ND	61.4
SB-0.5	147	8.01	114	237	0.29	129	ND	ND	ND	75.4
Quality standards	—	6–9	2.0	40	0.2	2.0	1.0	0.01	0.7	250 *
Discharge standard	—	6–9	15	100	0.5^a^	—	1.0	0.1	0.1	—
**The sample name**	**Fe**	**Mn**	**Zn**	**Cd**	**Pb**	**Hg**	**As**	**Cu**	**NO_3_^−^**	**Cl^-^**
	**mg·L^−1^**	**mg·L^−1^**	**mg·L^−1^**	**mg·L^−1^**	**mg·L^−1^**	**μg·L^−1^**	**mg·L^−1^**	**mg·L^−1^**	**mg·L^−1^**	**mg·L^−1^**
DB-0.5	0.85	0.300	0.018	ND	ND	0.04	0.0030	ND	3.95	224
DA-0.5	0.84	0.310	0.016	ND	ND	0.06	0.0035	ND	3.19	230
SB-0.5	0.89	0.304	0.018	ND	ND	0.05	0.0032	ND	3.20	225
Quality standards	0.3 *	0.1 *	2.0	0.01	0.1	1	0.1	1.0	10	250 *
Discharge standard	—	2.0	2.0	0.1	1.0	50	0.5	0.5	—	—

* Represents the standard limit value of surface water source for centralized domestic drinking water in the environmental quality standard for surface water, and a represents the phosphate concentration.

**Table 5:** Groundwater quality test results.

The Sample Name	pH	Turbidity	Chrominance	TDS	SO_4_^2-^	NH_4_^+^-N	Cl^-^	Total Hardness	Pb
	-	degree	times	mg·L^−1^	mg·L^−1^	mg·L^−1^	mg·L^−1^	mg·L^−1^	mg·L^−1^
G2	11.22	1.05 × 10^4^	16	1.30 × 10^3^	12.5	15.0	184	414	0.21
G3	7.86	31	8	738	101	0.258	160	285	ND
GW-standard	5.5–6.5; 8.5–9.0	10	25	2000	350	1.5	350	650	0.50
**The sample name**	**Cu**	**Zn**	**Na**	**Fe**	**Mn**	**Volatile Penol**	**sulfide**	**LAS**	
	**mg·L^−1^**	**mg·L^−1^**	**mg·L^−1^**	**mg·L^−1^**	**mg·L^−1^**	**mg·L^−1^**	**mg·L^−1^**	**mg·L^−1^**	
G2	ND	ND	130	ND	ND	0.0808	0.042	0.11	
G3	ND	ND	71.9	ND	0.034	0.0019	0.013	0.06	
GW-standard	1.50	5.00	400	2.0	1.50	0.01	0.10	0.3	

**Table 6:** Comparison of the removal rate of COD_Cr_ and NH_4_^+^-N with the addition ratio of polyaluminum ferric chloride (PAFC).

PAFC Dosing Ratio	0	0.4‰	0.7‰	1‰
Concentration of COD (mg·L^−1^)	274.78	252.32	205.56	176.03
Removal rate of COD(%)	--	8.17%	27.74%	35.94%
Concentration of NH_4_^+^-N (mg·L^−1^)	132.69	130.33	128.96	123.28
Removal rate of NH_4_^+^-N (%)	--	1.78%	5.07%	7.09%
